# Reproductive Effects of Endocrine Disruptors in Domestic Ruminants: Integrating In Vitro and In Vivo Evidence

**DOI:** 10.3390/ani15182712

**Published:** 2025-09-16

**Authors:** Vasiliki G. Sapanidou, Sophia N. Lavrentiadou, Maria P. Tsantarliotou

**Affiliations:** 1 Laboratory of Anatomy and Physiology of Farm Animals, Department of Animal Science, School of Animal Biosciences, Agricultural University of Athens, Iera Odos 75 Str., 11855 Athens, Greece; vsapanidou@aua.gr; 2 Laboratory of Animal Physiology, School of Veterinary Medicine, Faculty of Health Sciences, Aristotle University of Thessaloniki, 54124 Thessaloniki, Greece; slavrent@vet.auth.gr

**Keywords:** endocrine-disrupting chemicals, reproduction, ruminants, bisphenol A, per- and polyfluoroalkyl substances, bovine, sheep, oocytes, spermatozoa

## Abstract

Endocrine-disrupting chemicals (EDCs) have raised increasing concern due to their potential effects on reproductive health. This review focuses on the impact of EDCs, particularly bisphenol A (BPA) and its analogues, and per- and polyfluoroalkyl substances (PFAS), on domestic ruminants (cattle and sheep) by integrating findings from both in vitro and in vivo studies. The analysis highlights how exposure to EDCs affects steroidogenesis, oxidative stress responses, apoptosis, epigenetic regulation, and overall fertility markers, such as oocyte maturation, sperm motility, and embryo developmental competence. While most data originate from in vitro bovine studies, in vivo research in sheep offers valuable insights. Importantly, given the potential for EDCs to bioaccumulate in animal tissues, these findings hold significant implications for animal health, particularly regarding reproductive physiology and fertility rates.

## Simple Summary

Endocrine-disrupting chemicals (EDCs), such as bisphenol A, and per- and polyfluoroalkyl substances, present in pesticides and other products used in agriculture, are increasingly worrying because they resemble natural animal hormones and harm reproductive health in animal species. Cell-based research and in vivo studies in domestic ruminants (cattle and sheep) reveal that EDCs interfere with physiological processes by mimicking hormones and, thus, inflict harm on animal reproductive health in terms of irregular estrous cycles, reduced fertility, placental dysfunction, and compromised embryo development. Since EDCs can accumulate in animal tissues, these effects have a broader impact on livestock health and fertility.

## 1. Introduction

### 1.1. Definition and Classification of Endocrine-Disrupting Chemicals (EDCs)

Starling described “hormone” as a substance produced by glands that is released into the bloodstream and serves as a signal (inhibitory or stimulatory) to regulate physiological processes in distant organs. The definition of “hormone” has evolved significantly since its introduction in 1905. As biochemistry advanced, the definition of “hormone” included their chemical structure, leading to classifications based on their molecular composition [1]. By the late 20th century, scientific data suggested that hormones regulate gene expression and cellular functions, including metabolism and homeostasis. Today, the definition of “hormone” has become more integrative, considering the role of hormones in various physiological processes and their interactions with environmental pollutants. This observation highlights their complexity and significance in physiology and overall animal health [1,2].

The “endocrine disruptor hypothesis” suggests that certain synthetic chemicals, such as those present in pesticides, industrial by-products, and plastics derived from human activities, can interfere with the endocrine system [2]. These chemicals are called “endocrine-disrupting chemicals (EDCs)”. The International Program on Chemical Safety (IPCS) defined an EDC as “an exogenous substance or mixture that alters function(s) of the endocrine system and consequently causes adverse health effects in an organism or its progeny, or (sub)populations” [3]. EDCs can display different mechanisms of action, which include activation/inactivation of hormone receptors, stimulation/inhibition of hormone synthesis, alteration of signal transduction in hormone-responsive cells, and induction of epigenetic changes in hormone-producing or hormone-responsive cells [4].

Both in vitro studies and pharmacological studies in animal models revealed several characteristics of EDCs that render them biologically significant for animals. In a review article, Kim and Lee classified EDCs into five groups from a clinical point of view: persistent organic pollutants (POPs), non-persistent organic pollutants (non-POPs), heavy metals, air pollutants, and drugs [5].

Persistent-organic pollutants, including dioxins, polychlorinated biphenyls (PCBs), and polyfluoroalkyl substances (PFAs), are highly persistent and resist degradation, thus remain in the environment for exceptionally long periods of time [4]. Some of them have half-lives that exceed 10 years. They become widely distributed throughout the environment (soil, water and, most notably, air), they accumulate in the fatty tissue of humans and animals [6,7], and they are found at relatively high concentrations in the food chain [8,9]. POPs bioaccumulation is detrimental as these compounds are toxic to both humans and livestock [9,10].

On the other hand, non-POPs, including bisphenol A (BPA) and phthalates, have shorter half-lives [11,12], which, in combination with their low lipophilicity [11], reduces their bioaccumulation.

Both POPs and non-POPs have garnered scientific interest due to their ability to interfere with endocrine functions. Their potential to act as endocrine disruptors, along with their wide environmental distribution and accumulation in animal tissues (adipose tissue, blood serum, liver, brain) and products (milk, meat, eggs), render them a risk to “One Health”. It is now widely accepted that a vicious cycle of bioaccumulation is established, where animals are exposed to increasing concentrations of EDCs, which accumulate in their tissues and fluids, thus posing further risk to humans and animals that consume them. This constitutes a major concern for the One Health approach, thus emphasizing the need for integrative surveillance and mitigation efforts for EDCs to be mandatory. While POPs raise concerns due to their persistence and bioaccumulation [13], non-POPs raise alarm because of their widespread use and potential risks for animal health [14].

### 1.2. Main Sources of Exposure

Animals can be exposed to EDCs through various routes, including ingestion due to consumption of contaminated food and water, inhalation of air-borne pollutants, and absorption through the skin [8,9,15,16,17]. Ruminants are primarily exposed to EDCs by grazing or consuming feed grown on contaminated land. Agricultural runoff (the water that flows over farmland, picking up pollutants, is a major source of water pollution), polluted pastures, or feed derived from crops treated with pesticides can introduce EDCs through ingestion [18,19]. Moreover, manure can release endocrine-active substances into the environment, contaminating the soil and water near the farms. While it is widely accepted that diet and water are the primary exposure sources, the significance of other exposure pathways, such as inhalation, has not been extensively investigated and may yet prove to be important [20]. Regardless of the source of exposure, there are other factors that may also influence the concentration of EDCs in animal tissues and organs. These factors include (i) the chemical properties of the molecule (lipophilic EDCs tend to accumulate in fatty tissues [6,7]); (ii) the age and stage of development of the exposed animal (i.e., fetal, neonatal and adult [21]); (iii) the rate of pollutant uptake and subsequent degradation, excretion, and/or metabolism (for example in dairy cows, perfluorooctanoic acid (PFOA) has a higher elimination rate than perfluorooctanesulfonate (PFOS) [22]); and (iv) the composition of the diet (grazing in pastures fertilized with sewage sludge contaminated with different pollutants increases the risk of exposure and accumulation of EDCs in animal tissues [13,18]).

### 1.3. Relevance to Animal Reproduction, Productivity, and Health

The accumulation of EDCs in animal tissues can lead to various health issues, including hormonal and reproductive disorders. Hormonal disorders may affect puberty onset, cyclicity, prolificacy, and breeding efficiency. EDCs may also induce apoptosis and oxidative stress, alter gene expression, and disrupt cellular homeostasis, thereby increasing the risk of reduced cleavage and blastocyst formation rates, impaired embryonic developmental competence, and successful fertilization. The reproductive system of all animals is highly sensitive to hormonal fluctuations, making it particularly vulnerable to these environmental pollutants. Disruptions in the hypothalamic–pituitary–gonadal (HPG) axis, which regulates the release of key reproductive hormones such as gonadotrophins (luteinizing hormone (LH), follicle-stimulating hormone (FSH)], estrogens, androgens, and progesterone) can result in a range of adverse outcomes, including irregular estrous cycles, reduced fertility, and compromised embryo development [18,23,24].

As EDCs accumulate over time, the reproductive rates and productivity gradually decline [16,23]. Moreover, hormone disruption due to EDCs exposure causes alterations in thyroid function and metabolic processes that affect growth [25,26,27] and milk production due to impaired mammary gland development, lactogenesis, and suppressed endocrine signaling [28,29]. Furthermore, EDCs can have broader impact on animal health, as they suppress the immune system and, thus, make animals more susceptible to diseases [30]. Moreover, because EDCs affect neurodevelopment during prenatal and early postnatal development in humans, they influence behavior and potentially may affect social interactions and stress responses [31].

### 1.4. Aims and Scope of the Review

The list of chemicals recognized as having endocrine-disrupting properties is long, including approximately 1000 substances, such as pesticides, plasticizers, industrial chemicals, metals, pharmaceuticals, and phytoestrogens [4,25]. Several studies have investigated the effects of a plethora of endocrine-disrupting chemicals, such as phthalates, dioxins, and phytoestrogens [5,9,10], in humans and animal species. However, in order to restrain the length of this review, we decided to focus on BPA and PFAS for the following reasons:

- BPA and its analogues are among the most studied EDCs globally because of their widespread use in consumer products, despite regulatory restrictions in various regions, and their high persistence in the environment.
- BPA analogues are now widely used due to regulatory efforts for BPA substitution. However, recent data indicate that these analogues may be as harmful—or even more so—as BPA. The potential toxicity of BPA analogues has not been reviewed before.
- There is ample evidence of the endocrine-disrupting relevance to human reproduction: BPA and PFAS have an impact on estrogenic and androgenic pathways, steroidogenesis, Leydig cell function, and epigenetic regulation of reproductive capacity that is well-documented.

In this review article, we focus on the impact of PFAs, and BPA and its analogues, on the reproductive system of domestic ruminants (cattle and sheep), by integrating findings from both in vitro and in vivo studies. The literature search in the databases, using the selected keywords described in the Methodology Section (Section 1.5), yielded no relevant studies for goats. When there are no data on ruminants, important information from research with other species (humans, rodents) is provided, to provide a more complete overview of the topic.

The article provides a comprehensive framework by outlining all recognized endocrine-disrupting molecular mechanisms affecting ruminant reproduction and explores the pathways through which bisphenols (BPA and analogues) and PFAS exert their effects in vitro and in vivo. We anticipate that a review of these mechanisms will emphasize the need for strategies to mitigate the impact of these compounds and for practices to ensure the sustainability of ruminant farming, while also providing useful information.

### 1.5. Methodology

A literature search was conducted using Medline, PubMed, and Google Scholar databases, and was limited to papers published from 2010 to 2025. The following keywords and search terms were included: “BPA, BPS, BPF, bovine and sheep sperm”, “BPA, BPS, BPF, bovine and sheep oocytes”, “BPA, BPS, BPF, bovine and sheep embryos”, “PFAS, bovine and sheep sperm”, “PFAS, bovine and sheep oocytes”, “PFAS, bovine and sheep embryos”, “BPA, BPS, BPF, bovine and sheep pregnancy”, and “PFAS, bovine and sheep pregnancy”. The methodology is presented in Supplementary File S1. Only original research and review articles were included. Abstracts, short communications, and commentary articles were excluded. Articles that were retrieved by our search but were focused on a topic other than reproduction were also excluded. Also, we did not include in the review studies that, to our scientific opinion, had serious experimental design drawbacks (lack of proper controls, not suitable animal model, problematic experimental design).

The PRISMA flow diagram visually summarizes the systematic review’s study-selection process, mapping each stage from identification to inclusion. Initially, the total number of records identified through database searches was 5808. Following removal of duplicates, two of the authors (VGS and SNL) screened and evaluated the remaining articles (*n* = 2907) independently; initially, they screened the titles, the abstracts, and the keywords (*n* = 170), and subsequently proceeded to screen the full-text articles (*n* = 91). We included only articles that provide data on the effect of PFAS or BPA and its analogues on the reproductive system of sheep or bovine (as mentioned above, no data regarding goats have been retrieved) with a clear description of the sample size(s), the concentration(s), and dosage tested and the exposure route, before concluding to the studies included in this review article (*n* = 38).

## 2. Bisphenols and PFAS Exposure Pathways and Mechanisms of Action

### 2.1. Bisphenols

Bisphenols are used in the manufacture of plastics, such as polyvinyl chloride (PVC) and PCBs. Currently, BPA is the compound in the bisphenol group that is used in the highest volumes. Because it is highly toxic, and in order to reduce the potential risks, BPA is increasingly being replaced by other bisphenols, like bisphenol S (BPS) and bisphenol F (BPF). Unfortunately, current evidence indicate that these compounds are equally or even more toxic than BPA [32,33,34].

BPA is a plastic monomer present in epoxy resins, such as those used as a coating on food and beverage cans. When BPA polymers in plastic products are exposed to heat changes or acidic conditions, the monomers that link the ester bond of BPA can be hydrolyzed. The released free BPA monomers can leach into the environment and contaminate both soil, where they sediment, and water, where they are moderately soluble [35,36]. Animals are exposed to BPA from fetal life through adulthood, primarily through food, which is the most important source of exposure to BPA [19]. Other potential sources are air and dust [17]. BPA enters the atmosphere through human activities, such as incineration of BPA-containing materials, the trade of recycled or recyclable products, and accidental emissions near production facilities. In addition, indoor and outdoor dust acts as a significant reservoir, containing both free BPA and conjugated metabolites (e.g., sulfates, glucuronides), which are formed via biological metabolism [17]. Finally, BPA can be absorbed through the skin [15]. The drinking water pipes, and the rubber materials of milking machines, are coated internally with epoxy resins containing BPA, which can be hydrolyzed and leach over time, especially with aging and heat.

Exposure may also occur during assisted reproductive techniques (ARTs) via plastic consumables, such as plastic tubes, culture dishes, commercial media, and catheters [37]. Recent studies have demonstrated that commonly used plastic consumables leach BPA to the media at detectable concentrations (2–4 nM) [37,38] that are effective enough to cause spindle abnormalities in MII oocytes and chromosome misalignments [39,40,41]. ARTs for humans and animals largely make use of comparable plastic consumables, particularly in the collection, culture, and handling of gametes and embryos, which implies a similar risk of exposure.

BPA is metabolized by the liver [6]. In humans, unconjugated BPA—the biologically active form of BPA—is thought to be rapidly conjugated in the liver and then excreted via bile or urine, with a very short half-life of approximately 5.3 h [42]. However, the enzyme β-glucuronidase is present in detectable concentrations within organs, particularly the liver, kidney, ovary, and placenta in both animals and humans [43,44]. This enzyme is capable of deconjugating BPA and, thus, releasing its active, estrogen-disrupting form again [45]. This is of great significance during pregnancy, as conjugated BPA may cross the placenta, where, following deconjugation by the residing β-glucuronidase, can harm the fetus in utero [46,47].

BPS and BPF, like BPA, are also used in the production of epoxy resins and paper products. They, also, act as EDCs that mediate reproductive disorders by altering hormonal function and cellular responses [32]. Both analogues are now widely detectable in the population, thus raising concerns about their impact on reproductive outcomes [40,41,48]. BPF is more similar to BPA than BPS [49,50], which indicates that not all bisphenol alternatives are safer than BPA. This underlies the need for more, thorough research studies [33,34,51].

Bisphenols can act through different pathways to mediate their disrupting effects on the reproductive system:

- Disruption of normal cellular function by acting as estrogen [52,53], or androgen [54] antagonists. Thus, they can interfere with several physiological processes in reproductive tissues, such as gonad development and function, lactation, placental development, etc. BPA has structural similarities with estradiol (a phenol ring) and exhibits moderate estrogenic activity, despite a 10,000-fold lower binding affinity to estrogen receptors (ERs), compared to that of estradiol [55,56]. Thus, BPA is considered a weak estrogen due to its low affinity for nuclear estrogen receptors [11,55]. However, other studies have shown that BPA is equipotent with estradiol in its ability to initiate rapid nongenomic responses from membrane surface receptors.
- Reduction of steroidogenesis by granulosa and luteal cells [41,48,53,57] and production of anti-Müllerian hormone (AMH) by granulosa cells [58], thus interrupting oocyte growth and maturation.
- Overproduction of reactive oxygen species (ROS). BPA induces oxidative stress (OS) and decreases the expression of antioxidant enzymes, such as superoxide dismutase (SOD), catalase (CAT), and glutathione peroxidase (GPX). In bovine gametes, BPA and BPS differentially affect the activity of the above enzymes [51,59,60].
- Induction of apoptosis. BPA and BPF disrupt the balance between the proapoptotic BCL2-Associated X (BAX) and the anti-apoptotic B-cell lymphoma 2 (BCL-2) proteins promoting apoptosis [49].
- Epigenetic modifications. BPA regulates microRNA (miRNA) levels. For example, it increases the expression of miR-21, miR-155, and miR-29a, and decreases the expression of miR-34c and miR-10b [61,62,63]. These miRNAs play a regulatory role in bovine female fertility. In particular, increased expression of miR-155 impairs cumulus expansion and oocyte maturation, thus reducing cleavage and early embryo development rates [64]. Moreover, reduced levels of miR-34c have been associated with reduced blastocyst quality, as they negatively affect total cell number, inner cell mass (ICM) and ICM-to-total cell ratio [65].

BPA has been detected in human serum and follicular fluid at approximately 1–2 ng/mL (4.4–8.8 nM). It is also detected in fetal serum and full-term amniotic fluid, which verifies BPA passage through the placenta [66]. The median BPA concentrations in the maternal side of the placenta, umbilical cord plasma, placental tissue, and amniotic fluid, in humans are 19.0 nM, 8.0 nM, 22.2 nM, and 36 nM, respectively [66,67]. To the best of our knowledge, the concentration of BPA and its analogues in the reproductive tissues and fluids in ruminants have not been quantified.

### 2.2. PFAS

PFAS, also called “forever chemicals”, encompass a vast group of synthetic compounds characterized by carbon–fluorine bonds, which are responsible for their resistance to degradation [68] and their extreme thermal and chemical stability [69]. PFAS contain two main subgroups: PFOA and PFOS. The serum elimination half-time of PFOS in rodent species, monkeys, and humans is 25, 45, and 1970 days, respectively. The corresponding values of PFOA are ≤5, 14–42, and 1300 days, respectively [20]. PFAS are widespread in the environment, primarily due to their extensive industrial applications, such as in waterproof textiles and food packaging, and their resistance to degradation [69]. Animals are predominantly exposed to PFAS through water and food intake [22]. PFAS are metabolized by the liver, where they can accumulate, thus causing hepatoxicity, and are excreted from the body primarily through urine [70], while the mammary gland is an additional elimination route [71]. PFAS were also detected in the newborn and the placenta of humans and sheep, thus indicating transplacental transfer and potential negative reproductive outcome [46,72].

Two distinctive characteristics of PFAS are their amphiphilic character and high affinity to albumin, which allow their circulation in the body [73]. As a result, PFAS can cross barriers and can be detected in various organs, such as the brain, the testicle, and the ovary, in both humans and sheep [30,46,74].

PFAS mediate their disrupting effect on reproduction through two different processes:

- Disruption of normal cellular function by acting as agonists or antagonists of nuclear hormone receptors, including estrogen, androgen, and thyroid hormone receptors [75].
- Reduction of steroidogenesis by altering the expression of genes involved in steroid hormone synthesis (in bovine granulosa cells) [76].

However, it should be mentioned that the effect of PFAS depends not only on the type of PFAS, but also on the type of cell and the species. For instance, PFOS affects porcine theca cells but not granulosa cells, while PFOA has an impact on granulosa cells but not on theca cells [77].

In humans, concentrations of PFOS or PFOA over 20 ng/mL are considered toxic, leading to deficits in gamete function [78]. In males, exposure to PFAS may impair sperm motility and morphology, reduce testosterone levels, and inhibit capacitation/acrosome reaction, whereas in females it affects the oocyte competence leading to reduced cleavage and blastocyst rates [74]. The European Food Safety Authority (EFSA) has assessed the health risks associated with certain PFAS and recommended that blood plasma concentrations of PFOS, PFOA, perfluorohexane sulfonate (PFHxS), and perfluorononanoic acid (PFNA) should not exceed the guide value of 2.5 ng/mL [79,80].

## 3. Effects of EDCs on Reproductive Physiology in Ruminants

Animal exposure to EDCs during critical developmental periods can lead to permanent alterations in the endocrine system, affecting their reproductive health. Despite the known impact of EDCs on reproductive health, there is a lack of in vivo data on the effects of EDC exposure on ruminant fertility. Investigating the mechanisms through which EDCs exert their effects is essential for maintaining animal welfare and ensuring the economic viability of ruminant farming [81]. Moreover, ruminants, especially bovine, share notable physiological and reproductive characteristics with humans, including hormonal regulation and ovarian function. Moreover, the development of ARTs in bovine models allows for controlled studies on the effects of EDCs on gametes, embryos, and reproductive tissues [19,81]. While laboratory rodents offer valuable data in toxicological studies for the metabolism and kinetics of EDCs, ruminants may offer data more directly translatable to human health due to their longer lifespan and similarities with human reproductive physiology [82]. Ruminants provide a robust model for studying the endocrine and reproductive effects of EDCs, bridging the gap between controlled laboratory research, clinical trials, and real-world environmental exposures. Below, we discuss the effects of PBA, its analogues, and the most important PFAS on reproductive physiology of ruminants, which are summarized in Table 1, Table 2 and Table 3.

### 3.1. Effects of BPA In Vitro

#### 3.1.1. Effects on Oocytes

Exposure of oocytes to BPA during maturation negatively affects the reproductive outcome. In vivo, the lowest observable adverse effect level (LOAEL) of BPA in mice is 50 mg/kg/day and this concentration equates to 50 μg/mL of media, in vitro. On the other hand, the lowest concentration that causes a measurable adverse effect is 0.05 mg/mL (or 219 μM) [52]. This dose of BPA was used as a reference in a dose-dependent in vitro study with bovine oocytes, which verified that the cleavage and blastocyst rates in bovine were also negatively affected by BPA, compared to the control group [61]. Treatment of bovine oocytes with BPA at a concentration similar to the identified LOAEL in mice resulted in an uptake of 10.5 nM (2.48 ng/mL), which is similar to the concentration of BPA measured in human follicular fluid (up to 8.8 nM) [61,62]. Maturation of bovine oocytes in the presence of BPA reduced the blastocyst rate (19 ± 2% vs. 4.4 ± 0.8% for the BPA and control group, respectively) [61]. Moreover, exposure of ovine oocytes to BPS at the concentration detected in human follicular fluid (10 nM) did not affect oocyte viability, but the percentage of blastocyst rate was decreased by 34.4%, compared to the negative control [41]. Specifically, in this study, 10 nM BPS decreased the >4 cell embryo rate by 15.2%.

i. Effects of BPA at concentrations lower than LOAEL

Exposure of bovine oocytes to BPA during in vitro maturation (IVM) induced significant alterations in meiotic progression and spindle morphology. Specifically, exposure of oocytes to 30 ng/mL (130 nM) BPA notably decreased the percentage of oocytes reaching metaphase II (MII) by 15% [39], although only a small percentage of BPA was uptaken by the oocytes (the resulting average concentration of BPA measured in mature oocytes was only 2.48 ng/mL). Moreover, this exposure led to a substantial increase in spindle abnormalities (67.9%) and chromosome dispersal (60%). BPA mediated these effects even when tested at very low concentrations, as low as 1 fM [40].

Further investigations have demonstrated that exposure to 130 nM BPA during in vitro oocyte maturation skewed the sex ratio in bovine blastocysts, deviating from the expected 50:50 male-to-female ratio to a 34:66 ratio [83]. Interestingly, BPA did not alter the expression of several developmentally important genes, including the cell division cycle 2 (CDC2), aurora kinase A (AURKA), and p53 genes, in mature bovine oocytes and blastocysts. However, there was a significant increase in apoptosis and DNA damage in blastocysts derived from BPA-exposed oocytes, with apoptotic nuclei detected in both the inner cell mass and trophectoderm (TE) cells [83]. Conclusively, bovine oocytes are vulnerable to BPA exposure even at concentrations lower than the identified LOAEL in mice.

ii. Effects of BPA and its analogues at concentrations similar to the LOAEL in mice

Bovine oocytes are also adversely affected when exposed during IVM to 0.05 mg/mL BPA, a concentration similar to the LOAEL in mice. Specifically, BPA significantly reduced cleavage and blastocyst rates by approximately 40% (*p* = 0.0001) and 25% (*p* = 0.0015), respectively [58,59,61]. Incomplete cumulus expansion and darker cell cytoplasm were indicative of poor-quality cumulus–oocyte complexes (COCs) [62]. Interestingly, BPF exhibited similar effects, whereas BPS did not have an impact on cleavage and blastocyst rates [58,59]. These findings suggest that BPA and BPF, but not BPS, may share similar mechanisms of action.

The investigation of the underlying mechanisms revealed that BPA, but not BPS or BPF, induced the production of ROS in denuded bovine oocytes [59]. Additionally, these compounds have an impact on the antioxidant status of tissues. For example, both mRNA and protein expression of SOD2, GPX1, and GPX4 were decreased in BPA-treated COCs [59]. A deficiency in SOD2 or Glutathione Peroxidase (GPX) enzymes can lead to excessive ROS and cell damage, highlighting their interconnected roles in maintaining cellular redox balance. These enzymes form a coordinated antioxidant defense system against ROS, which interact with every cellular component, thus affecting the structural and functional integrity of the cells. Consequently, BPA-induced OS is a result of both ROS overproduction and alteration of antioxidant enzyme expression. Therefore, BPA has the capacity to disrupt the redox status within oocytes.

Bisphenols also affect developmental processes by disrupting cell communication and hormonal regulation during oocyte maturation. Both BPA and BPS dysregulate the expression profile of connexins in isolated cumulus cells [91] and impair steroidogenesis [48], while only BPA has an effect on COC characteristics. More specifically, bovine blastocysts derived from BPA-exposed COCs exhibited observable characteristics of low-quality embryos, including a collapsed ICM with fewer cells, a low percentage of expanded blastocysts, and improper TE organization. However, BPS did not have similar effects, suggesting that these two analogues probably act through different mechanisms, which involve cell communication through gap junctions and regulation by steroid hormones [48,91].

Moreover, BPA inhibited the production of AMH by bovine granulosa cells and induced the expression of its receptor AMHRII, suggesting a compensatory effect [58]. In the presence of BPA, the cleavage and blastocyst rates were compromised and embryos failed to reach the appropriate developmental stages in a timely manner, especially at the developmental stage of 2–4 and 8–16 cells, due to the promotion of apoptosis by AMH. The study also highlights that the protein expression of AMH (*p* = 0.0001) and AMHRII (*p* = 0.0011) was significantly upregulated in the BPA-treated groups. This upregulation correlates with the increase in DNA fragmentation (*p* = 0.0337) in the BPA-treated blastocysts [58]. Interestingly, a study on women undergoing IVF (in vitro fertilization) treatment has revealed that women with fertility problems present low serum concentration of AMH, correlated with high BPA concentration in the urine, compared to healthy women [92].

Furthermore, BPA exposure had a significant effect on the expression of specific miRNAs during oocyte maturation and early embryonic development. Notably, miR-21, miR-155, and miR-29a expression levels were markedly increased, while miR-34c and miR-10b expression levels were decreased in bovine BPA-treated COCs and embryos. These alterations in miRNA expression profiles may disrupt the regulation of genes that are critical for oocyte competence and embryonic development. In contrast, BPS exposure did not affect the expression of these miRNAs, providing further evidence of different mechanisms of action between the two compounds [61,62].

In conclusion, BPA can adversely affect oocyte quality and early embryonic development. These studies underscore its potential to disrupt critical molecular pathways involved in oocyte maturation, antioxidant defense, intercellular communication, hormonal signaling, and gene regulation. BPS, however, appears to have a distinct biological profile, with potentially less pronounced effects.

#### 3.1.2. Ovarian and Follicular Cells

Granulosa cells play a pivotal role in oocyte maturation by providing essential support for oocyte development and maturation, as well as by secreting factors that protect the oocyte. However, exposure to bisphenols can adversely affect granulosa cell function, leading to OS, apoptosis, and impaired steroidogenesis.

Recent studies have demonstrated that exposure to BPA and BPF, at concentrations corresponding to the LOAEL in mice, significantly reduced granulosa cell viability and increased ROS production within 12 h of culture. Antioxidant gene expression and protein levels were altered (increased SOD1 and CAT and decreased GPX4) [52]. Moreover, BPA and BPF induced apoptosis via the intrinsic mitochondrial pathway, as evidenced by increased expression of pro-apoptotic genes, such as BAX and caspase-9. In contrast, BPS did not exhibit significant apoptotic effects or activation of the intrinsic pathway under similar conditions [49]. Furthermore, the detrimental effects of BPA or BPF on bovine granulosa cells extend to steroidogenesis. Exposure of ovine granulosa cells to 10 μΜ and 50 μΜ BPA or BPS for 48 and 96 h decreased progesterone concentration [53], likely due to inhibition of steroidogenic enzymes, such as cytochrome P450 side-chain cleavage (CYP11A1) and steroidogenic acute regulatory protein [93]. Interestingly, only BPS decreased estradiol concentration, which implies that the two compounds act, at least partly, through independent mechanisms, in spite of their structural similarities [53]. In addition, exposure of bovine theca cells to BPA (100 μM) significantly reduced cell viability, which was accompanied by a reduction in progesterone production by these cells [33].

In conclusion, exposure to bisphenols adversely affects granulosa and theca cell function by inducing OS and apoptosis, and impairing steroidogenesis, which can compromise oocyte maturation and fertility.

#### 3.1.3. Spermatozoa

Exposure of fresh or cryopreserved bovine spermatozoa to BPA, BPS, and BPF affects their quality characteristics. Among them, BPA is considered the most harmful, BPF affects only cryopreserved spermatozoa, while BPS appears to be the least harmful, with no significant effects [34,63]. BPA (0.05 mg/mL) reduces sperm motility (*p* < 0.001) and viability (*p* < 0.01) and inhibits capacitation in both fresh and cryopreserved bovine sperm (*p* < 0.05) [34,63] by disrupting mitochondrial membrane potential and inducing OS [34,59], with frozen/thawed spermatozoa being more vulnerable [33]. Moreover, Lukacova and colleagues exposed fresh bovine spermatozoa to lower concentrations of BPA (0.001 mg/mL, 0.01 mg/mL, 0.1 mg/mL, and 0.2 mg/mL) and concluded that BPA effects are dose- and time-dependent and involve an increase in superoxide anion levels [84]. In particular, sperm motility decreased immediately (42% vs. 92% for the 0.2 mg/mL BPA and control group, respectively) after the addition of high concentrations (>0.1 mg/mL) of BPA, whereas lower concentrations (>0.01 mg/mL) decreased sperm motility after 6 h (67.33% vs. 73.88%, for 0.1 mg/mL BPA and control group, respectively). Viability was decreased in all groups, with significant differences noted only at the highest doses (0.1 mg/mL and 0.2 BPA mg/mL) after 24 h (37.99% and 29.20% vs. 100%, for 0.1 mg/mL, 0.2 BPA mg/mL and control group, respectively). The observed decline in sperm quality was attributed to overproduction of ROS, as BPA increased OS in thawed bovine spermatozoa [59,84]. In conclusion, BPS and BPF might be less damaging for spermatozoa compared to BPA, probably because they do not induce the same level of OS to male gametes.

Bisphenols also affected the capacitation status of fresh and frozen/thawed bovine spermatozoa. Exposure of spermatozoa to high concentrations of BPA or BPF inhibited capacitation, compared to the control, possibly through a decrease in tyrosine phosphorylation, while BPS had no significant effects [34,63]. Treatment of fresh or frozen/thawed bovine spermatozoa with BPA also decreased their viability and increased the number of apoptotic and necrotic spermatozoa, as determined by the decreased mitochondrial membrane potential [34]. Davis and colleagues [63] also tested how BPA or its analogues affect the fertilizing capacity of fresh or cryopreserved bovine spermatozoa and the miRNA profile of spermatozoa and embryos. The exposure of spermatozoa to BPA significantly reduced cleavage and blastocyst rates, compared to the control group. The quality of the embryos was also affected, since the blastocysts derived from BPA-, BPS-, and BPF-treated sperm had significantly fewer total cells, TE cells, and ICM cells and higher DNA fragmentation index. No statistically significant changes in miRNA levels were observed in sperm or blastocysts, although trends were noted in this study (e.g., decreased miR-93 in BPS-treated blastocysts).

#### 3.1.4. Embryos

Choi and co-authors [85] studied the effects of acute BPA exposure (10 ng/mL) on bovine embryos between days 3.5 and 7.5 post-fertilization. They found that BPA reduced blastocyst development and embryo quality, without affecting cell number. Embryos exposed to BPA showed altered metabolism and increased glucose consumption. These effects were similar to those caused by estradiol and were reversed by an ER inhibitor (fluvestrant), thus supporting BPA’s action through estrogen-modulated pathways. BPA also causes mitochondrial disruption, impairs embryonic development, and leads to embryo arrest or loss. Moreover, mitochondrial and metabolic disruption can have long-term consequences on individuals, such as insulin resistance and obesity [94]. Overall, exposure of embryos to BPA during early embryo development can affect their growth, quality, and metabolism, primarily through estrogen-mediated mechanisms, though other pathways cannot be ruled out.

### 3.2. Effects of BPA In Vivo

Farm animals are exposed to relatively high concentrations of EDCs because these chemicals persist in the environment (water, soil, air). EDCs accumulate in the fat tissue [6,7] and when fat is mobilized during pregnancy or lactation, EDCs are released and, thus, affect embryos and neonates, which are thus exposed to them at relatively high concentrations [46,47,64]. The metabolic processing of EDCs remains poorly understood; however, studies have demonstrated that interspecies variability in the activity of certain enzymes responsible for transforming and detoxifying these substances can exceed 1000-fold. The disparity is evident in how both exogenous and endogenous compounds influence liver function across different species [95]. In ruminants, the degradation and absorption of EDCs may be affected more significantly by the microbial populations in the rumen than by hepatic biotransformation processes [6]. Nevertheless, there is clearly a risk of significant bioaccumulation and its consequent effects on health and reproductive outcome. Currently, the majority of the in vivo studies use sheep as models to study the potential risks of EDCs. We can speculate that the use of bovine is limited due to the prohibitive cost of housing and feeding these animals, their long gestation periods (approximately 280 days), and extended developmental timelines before reaching maturity. Therefore, the bovine model remains valuable for in vitro studies, as mentioned above.

The effects of BPA on placental efficiency were studied in sheep. Daily subcutaneous injections of BPA (0.5 mg/kg/day) from gestation day (GD) 30 to GD 90 resulted in significant reductions in fetal weight and placental efficiency on GD 65 (*p* = 0.04), with no significant changes on GD 90 [86]. Gestational BPA exposure negatively affected placental function through various mechanisms, including the disruption of oxidative balance (*p* < 0.001) and steroid hormone action (*p* < 0.001) on GD 90 and GD 65, respectively. The study verified the stimulating effect of BPA on ERs, since BPA upregulated the expression of ER type 2 (*p* < 0.001) on GD 65, with no impact on androgen receptor (AR) expression. Moreover, BPA exposure during the gestation period altered the inflammatory profile of the placenta with increased expression of interleukin 8 (IL-8) (*p* = 0.005) and decreased mRNA expression of tumor necrosis factor alpha (TNF-α) (*p* = 0.04) on GD 65. BPA induced LPO and upregulated the expression of the antioxidant enzymes glutathione reductase-GSR (*p* = 0.001), SOD1 (*p* = 0.007), and SOD2 (*p* < 0.01) on GD 65. These enzymes comprise an enzymatic antioxidant defense system of cells and tissues, placenta included. SOD1 and SOD2 convert damaging superoxide radicals into hydrogen peroxide, while GSR regenerates reduced glutathione, ensuring the glutathione-based detoxification pathway remains functional. Their upregulation in response to BPA exposure likely reflects the cooperation of different antioxidant compounds to counteract the oxidative stress in placental tissue. In another study, Zhang and colleagues [60] employed a higher dose of BPA (5 mg/kg/d, sc) for a longer period (GD 40–GD 130). This exposure resulted in reduced fetal weight, placental efficiency, and progesterone concentration (*p* < 0.05) and was significantly correlated with OS, as evidenced by increased levels of ROS and LPO (*p* < 0.05). Moreover, BPA-induced placental dysfunction correlated with mitochondrial dysfunction, activation of the intrinsic mitochondrial apoptosis pathway, and autophagy in placental cells (*p* < 0.05). The role of endoplasmic reticulum stress (ERS) as a mediator in BPA-induced placental injury was also highlighted. Activation of ERS exacerbated OS, apoptosis, inflammatory responses, autophagy, and placental dysfunction, suggesting that inhibition of ERS could mitigate these effects and offer potential therapeutic targets for managing BPA-induced pregnancy complications.

When evaluating in vivo the impact of long-term dietary exposure to bisphenols, one has to take into consideration the metabolic status of the organism studied. An in vivo study in ewes with different metabolic statuses (restricted vs. well-fed groups) demonstrated that chronic (at least 3 months) BPS exposure (50 μg/kg/day per os) increased estradiol concentrations specifically in the preovulatory follicular fluid of well-fed ewes (*p* = 0.020), suggesting an estrogenomimetic action of bisphenols via their interaction with both nuclear and membrane ERs [96]. This elevation in estradiol may result from enhanced expression and activity of aromatase CYP19A1 in the ovary or reduced hepatic metabolism of estradiol. CYP19A1 is a key enzyme responsible for converting androgens into estrogens in ovarian granulosa cells that its mRNA expression in is upregulated by BPS [87]. In contrast, following exposure to BPS, restricted ewes exhibited decreased plasma concentration of progesterone (1.4-fold lower), estradiol, and estrone, compared to well-fed ewes (*p* = 0.007); notably, these hormonal disruptions varied across tissue compartments, indicating that BPS exerts cell-specific effects on steroidogenesis [96].

In conclusion, BPA exposure during gestation adversely affects placental function and fetal development in sheep. The mechanisms underlying these effects involve OS, hormonal disruption, inflammatory responses, mitochondrial dysfunction, autophagy, and endoplasmic reticulum stress. The role of EDCs in the pathophysiology of OS and the related diseases, infertility included, should be taken into consideration.

### 3.3. Effects of PFAS In Vitro

The data regarding the effect of PFAS on reproductive physiology of ruminants are as yet limited. In the work performed by Hallberg et al., the concentrations of PFAS that were used were chosen according to preliminary experiments performed by the research group, and the concentration of PFAS detected in human serum and follicular fluid [88,89]. The presence of low concentrations of PFOS (4 nΜ) in IVM media (similar to the concentrations found in follicular fluid) had no effect on cleavage rate and early development. Only a high concentration of PFOS (106 nM), which, according to the authors, “is within the range of human relevance”, reduced the proportion of cleaved oocytes to 0.76 vs. 0.83 in the control group (*p* = 0.04), and the proportion of those cleaved beyond the 2-cell stage to 0.53 vs. 0.63 in the control group (*p* = 0.01), with no effect on further stages. Although the production of blastocysts on day 8 was not affected, PFOS at this concentration delayed development of blastocysts by day 8 into more advanced stages, as evidenced both by a reduced developmental stage and a reduced number of blastomeres on day 8 (*p* = 0.04). Interestingly, the lipid distribution in the blastocysts treated with PFOS (106 nM) was altered, with increased total lipid volume in the embryo (853 μm^3^ vs. 607.6 μm^3^ in the control group, *p* = 0.0003) and increased lipid volume per cell (61,543 μm^3^ vs. 53,371 μm^3^ in the control group, *p* < 0.0001) [88].

This disturbance is indicative of disturbed metabolism, high risk of LPO, and compromised developmental potential, thus leading to apoptosis and reduced cryotolerance [97]. Gene expression analysis revealed that pathways related to differentiation, cell death, and survival were negatively affected. Similar observations were reported for PFHxS, which has been increasingly used after the phase-out of PFOS [89]. The exposure of oocytes to PFHxS at concentrations ranging from 0.01 mg/mL to 0.1 mg/mL was accompanied by a decrease in cleavage rates and disruption of early embryonic development (*p =* 0.04). Lower concentrations did not affect embryo developmental competence. On the contrary, the exposure of COCs to PFHxS concentrations ≥ 0.04 mg/mL resulted in a decreased number of cleaved embryos and cleaved beyond the two-cell stage, 44 h post IVF (*p* = 0.02). Importantly, there was no reduction in the proportion of embryos developed compared to the control, which indicates that the observed changes are more likely attributed to decreased developmental competence rather than early embryo loss. PFHxS concentrations ranging from 0.001 to 0.01 mg/mL disturbed embryo metabolism and altered lipid distribution, which was characterized by increased total lipid volume and lipid volume per cell (*p* = 0.01). Among these, transcriptomic analysis showed an upregulation in pathways related to increased lipid synthesis and production of ROS in 8-day post-fertilization embryos and inhibition of estrogen-activated pathways [89]. These results are in line with previous findings in a bovine model where the exposure of COCs during IVM to 0.01 mg/mL PFNA, another PFAS with potentially disrupting properties, can disrupt lipid metabolism in the developed embryos, even at concentrations that do not affect overall development [90].

The negative impact of PFAS on the developmental competence of oocytes can be partially attributed to the inhibition of steroidogenesis and mitochondrial dysfunction in bovine granulosa cells, which control the follicular microenvironment through the secretion of multiple growth factors and hormones [76,77]. For instance, PFOA (40 μΜ) decreased mRNA and protein levels of key steroidogenic enzymes, including CYP11A1, HSD3B, and CYP19A1, in bovine granulosa cells, verified by the suppressed levels of progesterone and estradiol [76]. Other potential mechanisms suggested by Hallberg and colleagues implicate the elevated levels of ROS and the subsequent OS, apoptosis, and autophagy [90].

PFOS exposure impairs key developmental signaling pathways, such as CTNNB1 (β-catenin/WNT signaling) [88], which is implicated in proper blastocyst morphology [98], or the RELA/NF-κB pathway [88], which is responsible for development past the two-cell stage [99]. Inhibition of the latter pathway is associated with reduced cleavage competency. Exposure to PFOS and PFNA is also accompanied by increased lipid accumulation, a response to embryonic stress and metabolic dysregulation [88,90]. The cryotolerance of embryos is then compromised [97]. Moreover, the exposure of bovine oocytes to PFHxS during in vitro maturation produced a dose-dependent range of deleterious effects on subsequent embryonic development [89]. High concentrations (≥40 μg/mL) of PFHxS significantly reduce the developmental competence, while exposure to concentrations between 1 and 10 μg/mL disrupt lipid distribution within blastocysts. Finally, at lower concentrations (0.1 µg/mL) ROS are overproduced, along with activation estrogen signaling pathways. Consequently, the blastocysts produced were characterized by DNA methylation alterations in blastocyst-stage embryos [89]. Although in vitro studies clearly demonstrate that PFAS exhibit distinct toxicodynamic profiles, these findings gain even greater significance when considering that multiple PFAS compounds coexist in the environment and bioaccumulate in tissues. In this context, animals that are not exposed to individual PFAS, but rather to complex mixtures of these compounds, may be affected in ways not fully represented by single-compound experiments in terms of reproductive physiology.

## 4. The Potential for Cumulative or Synergistic Impacts of BPA and PFAS in Ruminants

BPA and PFAS share overlapping mechanisms of toxicity, but they do exhibit distinct characteristics due to their diverse chemical characteristics and interactions with the reproductive system. They both bind to estrogen and androgen receptors, potentially activating or inhibiting estrogenic and androgenic responses. This interaction can disrupt steroidogenesis by altering the expression of genes involved in steroid hormone synthesis [48,53,57,76,77,96]. BPA and PFAS induce OS that leads to cell damage [49,51] and cause epigenetic modifications [63]. BPA alters microRNA expression profiles [61,63], which can affect protein expression and cell survival. On the other hand, exposure to PFAS has been shown to induce DNA methylation changes in genes related to stress response pathways [62,87]. Given their co-occurrence in the environment and in animal tissues, there is a strong possibility that in animals, we observe their synergistic or cumulative effects.

Indeed, co-exposure studies reveal complex interactions. Human perinatal stem cells exposed to both bisphenols and PFAS compounds (0.1 μM BPA, BPS, PFOS, and PFOA) exhibit mitochondrial dysfunction, reduced membrane potential, and evidence of mitophagy—a cascade not observed when cells are exposed to individual substances separately [100]. Moreover, the simultaneous exposure of pregnant rats to BPA (100 μg/L) and PFOS (2000 μg/L) through the drinking water led to an increase in interventricular septal thickness of approximately 20%, indicative of cardiotoxicity. This effect was attributed to increased cardiomyocyte size and a concomitant increase in collagen content. Interestingly, in vitro experiments in the same study suggest that these effects are rather independent [101]. Currently, while it is generally accepted that combined exposure to BPA and PFAS can lead to enhanced toxicity compared to individual exposures, the data are rather conflicting and non-consistent and the observed effects are not always additive or synergistic [98].

Nevertheless, the observations support a hypothesis that BPA and PFAS co-exposures could synergistically amplify oxidative stress, mitochondrial impairment, hormonal disruption, and epigenetic reprogramming—especially within the reproductive context. Although data in ruminants are currently lacking, there are ample data in humans and rodents that could provide useful information to guide future research focus in ruminants. Undoubtably, the data underscore the need for further research in livestock systems, and ruminants in particular, to investigate the effects of co-exposure to these compounds and to assess One Health risks more accurately.

## 5. Limitations

This review aims to provide a concise summary of all current data on the pleiotropic effects of EDCs on the reproductive system of ruminants. We have gathered all information on ruminant species and attempted to critically compare it with data in monogastric species. The review highlights not only the mechanistic pathways of two high-impact chemical groups—bisphenols (BPA and analogues) and PFAS—but also the important research gaps that exist in the field. Research that will provide knowledge to fill these gaps will significantly deepen our future understanding of the relationship between endocrine-disrupting chemicals and reproduction. As anticipated, this effort is impeded by several limitations. A rather important limitation is the absence of published data that report at what concentrations BPA and PFAS are encountered in the follicular fluid of ruminants. Available data are almost exclusively derived from human studies (e.g., women undergoing IVF), rather than from the ovarian/oviductal microenvironment of livestock. While the presence of PFAS has been documented in ruminant serum, liver, kidney, muscle, and milk, these tissues do not reflect the environment where the oocyte/zygote is exposed to during its development [22]. The observed concentrations of PFAS in blood and tissues, following exposure of animals to feed experimentally contaminated with perfluoroalkyl acids (PFAA) and the range of concentrations of BPA in the blood of buffaloes from different farms, are presented in Table 4 [22,102]. Notably, there exists considerable heterogeneity in experimental protocols—such as the duration of exposure, PFAS concentration in the feed, the age of the animals, and their breeds—which severely limits the comparability of the results across studies. This is particularly acute in the case of sheep; the available studies encompass only a small number of animals per study and exhibit substantial variability in their outcomes [103]. Moreover, BPA is found in buffalo blood serum at concentrations ranging from 0.7 to 28 nM—a wide range, indicating variable exposures likely via feed or environmental contamination [103]. In addition, BPA levels detected in milk can also significantly vary, according to the geographical area, the type of milk product (liquid, powdered), and the packaging material [104].

In terms of in vivo studies, the BPA/BPS dosages used in vivo, particularly the higher subcutaneous (sc) dose of 5 mg/kg, may not reflect typical environmental exposure. While administration of low doses, such as 0.5 mg/kg sc, have been shown to achieve fetal BPA levels comparable to those reported in human pregnancy cohorts (and are thus considered environmentally relevant) [86], the higher (5 mg/kg) dose is considered pharmacological. Such elevated doses are commonly employed in experimental toxicological studies to elicit measurable effects, facilitate dose–response assessments, and explore potential mechanisms of action [100]. Similarly, in the case of dietary BPS exposure, the 50 μg/kg/day dose used by Téteau and colleagues [96] was selected based on the EFSA 2015 guidance on BPA exposure limits in food. Nonetheless, caution must be exercised when extrapolating findings from these high-dose models to real-world exposure scenarios, as they may not fully replicate the biological responses to chronic low-level exposures that are typically encountered in the environment.

Another important limitation is that the materials commonly used in ARTs for animals are almost never tested for the presence of BPA or its analogues. Therefore, we have not quantified the potential “intrinsic” exposure sources within the experimental system.

Finally, we must note that several of the articles—particularly those related to the in vitro effects of BPA—have not been validated through independent, cross-laboratory replication. At present, these results stem from one single research group and, thus, their repeatability and general applicability remains to be tested by other groups, in other experimental systems.

## 6. Proposed Biomarkers to Assess the Toxicity of EDCs

There is indeed increasing interest on EDCs from the perspective of animal health. Beyond the aspects already discussed, it is particularly valuable to establish specific indicators/biomarkers that can reliably reflect the impact of EDCs on animal health.

i. BPA Concentration in Biological Matrices

The concentration of BPA and its analogues in urine provides a direct biomarker of exposure. Several bisphenols have been quantified in the urine of bovine species, supporting the utility of this non-invasive method. The mean concentration of bisphenol analogues in bovine urine is 59.6 ng/mL [105]. Moreover, hair samples have been validated as a reliable biomonitoring matrix in dairy cows, with the use of liquid chromatography–mass spectrometry (LC-MS) to determine the concentration of BPA [106]. Using this method, the median level of BPA detected is approximately 40.7 nM, suggesting that hair is a practical option for assessing long-term exposure. The effect of BPA and its analogues can be also assessed indirectly through their endocrine disrupting effects. For example, many studies indicate the implication of BPA in the pathophysiology of OS. Thus, the evaluation of the oxidative status of animals may also be a potential biomarker. Moreover, BPA has been shown to interfere with thyroid hormone receptor activity and disrupt thyroid signaling pathways. As a result, changes in levels of thyroid-stimulating hormone (TSH), triiodothyronine (T3), and thyroxine (T4) can serve as useful tools to demonstrate endocrine disruption [75,107]. Reproductive hormones may not constitute reliable biomarkers, as their levels are subject to physiological fluctuations throughout the estrous cycle. Studies in mice have shown that BPA exposure may also alter key serum parameters, including glucose, total protein, and albumin level [108]. Similar alterations in ruminants could provide early evidence of BPA-induced physiological stress.

ii. Concentration of PFAS in biological matrices

Several PFAS have been detected in the serum, milk, and muscle tissues of ruminants. However, plasma and skin (ear notch) samples have been used effectively in cows exposed to PFAS making skin sampling particularly a valuable tool to assess long-term exposure when blood collection is impractical or when a non-invasive technique is required [109]. The evaluation of kidney and liver function may also reflect the effect of PFAS exposure. Elevated levels of gamma-glutamyl transferase (GGT), changes in the albumin/globulin ratio, and alterations in biochemical markers, such as total CO_2_, creatine kinase (CK), and calcium, have all shown correlations with serum PFAS concentrations in domestic dogs and horses [110]. Moreover, disruptions in cholesterol and lipid metabolism have been reported, largely attributed to PPARα-mediated effects in the liver, indicating that serum lipid profiles may serve as sensitive indicators of PFAS-related metabolic stress [111]. Finally, metabolomic and transcriptomic analyses have yielded promising biomarker candidates. Specifically, PFAS exposure has been associated with disrupted bile acid synthesis and lipid metabolism pathways, reflected by elevated levels of glycerophospholipids, ceramides, triglycerides, and changes in specific fatty acids, such as oleic acid [112]. Additionally, transcriptomic profiling in mouse liver has shown upregulation or downregulation of genes involved in lipid metabolism, cholesterol regulation, and cellular proliferation—including Fatty Acid Synthase (Fasn), Proprotein Convertase Subtilisin/Kexin Type 9 (Pcsk9), and Cyclin D1 (Ccnd1) [113]. Therefore, it should be emphasized that the abovementioned associations remain hypothetical, and further laboratory and clinical investigations are necessary to validate the correlations among them.

## 7. Conclusions and Future Directions

The data reviewed in this article underscore the potential of EDCs to pose a risk against ruminant fertility and have a negative impact on reproductive physiology, advocating for further investigation. Despite the extensive research over the past 15 years, the data, particularly those concerning PFAS, are still limited. Moreover, current animal studies often use concentrations and chemical mixtures that may not be environmentally or tissue relevant, thus limiting the applicability of findings to natural conditions. Regarding ruminants, species-specific toxicokinetic studies will be very valuable, as EDCs exhibit significant interspecies variability in absorption, distribution, metabolism, and excretion profiles, which influence their effects. Such differences can lead to varying concentrations in animal tissues and, consequently, distinct toxic responses. Understanding these species-specific differences is essential for extrapolating data from animal models to humans and for developing accurate risk assessments for each species. Moreover, these variations underscore the necessity for tailored regulatory approaches and the consideration of species-specific data in environmental health policies. Longitudinal in vivo studies should be addressed to understand the long-term health impacts, as these “forever chemicals” accumulate in the body. Finally, the presence of both BPA and PFAS in the environment and their potential cumulative or synergistic effects raise concerns for livestock reproductive health. These chemicals can interfere with hormonal signaling pathways that may affect puberty onset, cyclicity, prolificacy, and breeding efficiency. Therefore, understanding the combined impact of these substances is crucial for developing strategies to mitigate their effects on reproductive health.

## Figures and Tables

**Table 1:** The in vitro effects of BPA and its analogues on reproductive physiology of ruminants.

Cell Type	Dose	Effects	References
Bovine oocytes	0.03 mg/mL BPA	Skewed sex ratio (34 male: 66 female) ↑ apoptosis and DNA damage in blastocysts	[83]
	0.03 mg/mL BPA	↓ % oocytes in MII ↑ in spindle abnormalities	[39,40]
Bovine oocytes	0.05 mg/mL BPA	↓ AMH production ↑ AMHRII expression	[58]
	0.05 mg/mL BPA and BPS	↓ activity of enzymatic antioxidants ↓ of oocytes reaching MII ↓ fertilizing capacity ↓ cleavage and blastocyst rates	[59]
COCs and embryos	0.05 mg/mL BPA	↑ expression miR-155 and miR-29; ↓ expression miR-34c and miR-10b	[61,62,63]
Ovine oocytes	0.05 mg/mL BPS	↓ % oocytes in MII and blastocyst rates no effect on viability	[41]
Bovine spermatozoa (fresh)	0.05 mg/mL BPA	↓ motility, viability and mitochondrial potential inhibited capacitation, ↑ ROS	[34,59,63,84]
Bovine spermatozoa (cryopreserved)	0.05 mg/mL BPA and BPF	Inhibited capacitation ↓ viability, ↑ apoptosis and necrosis	[34,59,63]
Bovine embryos	0.01 mg/mL BPA	↓ blastocyst development and embryo quality ↑ lipid accumulation	[85]
Bovine granulosa cells	0.05 mg/mL BPA and BPF	↓ viability, ↑ apoptosis ↑ ROS altered antioxidant genes expression	[49,51]
Ovine granulosa cells	10–50 μM BPA and BPS	↓ progesterone (BPA and BPS) ↓ estradiol (PBS) disruption of gene expression	[53]

↑: increase, ↓: decrease.

**Table 2:** The in vivo effects of BPA and its analogues on the reproductive physiology of ruminants.

Species	Dose	Effects	Reference
Sheep	0.5 mg/kg/day BPA subcutaneous injection GD 30–GD 90	↓ fetal weight, placental efficiency ↑ ROS altered hormone receptor expression	[86]
	5 mg/kg/day BPA subcutaneous injection GD 40–GD 130	↓ fetal weight, placental efficiency, progesterone levels ↑ ROS; mitochondrial dysfunction	[60]
Sheep	50 μg/kg/day BPS oral chronic (3 months)	↑ estradiol in preovulatory follicular fluid ↓ hormone concentration in metabolically restricted ewes	[87]

↑: increase, ↓: decrease.

**Table 3:** Effects of PFAS on bovine oocyte quality and embryo development in vitro.

Cell Type	Compound	Dose	Effects	Reference
Oocytes	PFOS	53 ng/mL (106 nM)	Delayed cleavage to the two-cell stage at 44 h post-fertilization ↓ number of blastomeres and altered lipid distribution ↓ developmental potential and cryotolerance	[88]
	PFHxS	0.01–0.1 mg/mL	↓ cleavage rates and disruption of early embryonic development (≥0.04 mg/mL) ↑ total lipid volume and lipid volume per cell ↑ ROS production, and inhibition of estrogen-activated pathways	[89]
Granulosa cells	PFNA	0.01 mg/mL	Disrupted lipid metabolism in developed embryos (even at concentrations not affecting overall development)	[90]
	PFOA	40 μM	↓ mRNA and protein levels of key steroidogenic enzymes (CYP11A1, HSD3B, CYP19A1) ↓ progesterone and estradiol ↑ ROS, apoptosis, autophagy	[90]

↑: increase, ↓: decrease.

**Table 4:** Concentrations of PBA ^#^ and PFAS * in ruminant tissues and fluids.

Matrix	BPA	PFOA	PFOS	PFHxS
Blood serum/Plasma	0.7–28 nM	~0.12–0.58 nM	~0.12–0.58 nM	0.025–1.625 nM
Milk (Group 1)		11.3 nM	4.65 nM	0.032 nM
Milk (Group 2)		34.0 nM	4.37 nM	0.013 nM
Muscle (Group 1)		1.67 ± 0.68 nM	289.9 ± 72.0 nM	47.7 ± 17.7 nM
Muscle (Group 2)		~0.08 ± 0.14 nM	356.0 ± 96.0 nM	12.2 ± 6.7 nM

* Values were retrieved from a feeding study [22]. Cows were fed on a feed that was contaminated with PFAA for 28 days. Those in Group 1 were slaughtered directly after the PFAA-feeding period (day 29), whereas animals in Group 2 were fed a PFAA-free feed for another 21 days (depuration period) before slaughter on day 50. ^#^ Values retrieved from a study with buffaloes from different farms [102].

## Data Availability

No new data were created or analyzed in this study. Data sharing is not applicable.

## Supplementary Materials

The following supporting information can be downloaded at: https://www.mdpi.com/article/10.3390/ani15182712/s1, File S1: PRISMA Flow Diagram. Reference [114] is cited in the Supplementary Materials.

## Abbreviations

The following abbreviations are used in this manuscript:

AMH	anti-Müllerian hormone
AMHRII	anti-Müllerian hormone receptor II
ARTs	assisted reproductive techniques
AURKA	aurora kinase A
BCL-2	B-cell lymphoma 2 protein
BAX	BCL2-associated X protein
BPA	bisphenol A
BPF	bisphenol F
BPS	bisphenol S
CAT	catalase
CDC2	cell division cycle 2
COCs	cumulus–oocyte complexes
CYP19A1	cytochrome P450 family 19 subfamily A member 1
CYP11A1	cytochrome P450 side-chain cleavage
EDCs	endocrine-disrupting chemicals
EFSA	European Food Safety Authority
ERS	endoplasmic reticulum stress
ER	estrogen receptor
FSH	follicle-stimulating hormone
GD	gestation day
GPX	glutathione peroxidase
GSR	glutathione reductase
HPG axis	hypothalamic–pituitary–gonadal axis
ICM	inner cell mass
IPCS	International Program on Chemical Safety
IVM	in vitro maturation
IVF	in vitro fertilization
LH	luteinizing hormone
LPO	lipid peroxidation
LOAEL	lowest observed adverse effect level
miRNA	microRNA
non-POPs	non-persistent organic pollutants
OS	oxidative stress
PFOA	perfluorooctanoic acid
PFOS	perfluorooctyl sulfonate
PFHxS	perfluorohexane sulfonate
PFNA	perfluorononanoic acid
POPs	persistent organic pollutants
PCBs	polychlorinated biphenyls
PFAS	polyfluoroalkyl substances
PVC	polyvinyl chloride
ROS	reactive oxygen species
SOD	superoxide dismutase
TE	trophectoderm
