# Supplementary material for: Reproductive Effects of Endocrine Disruptors in Domestic Ruminants: Integrating In Vitro and In Vivo Evidence
Source: Animals (Basel). 2025 Sep 16;15(18):2712. doi: 10.3390/ani15182712 (PMC12466432; doi:10.3390/ani15182712)
Supplement: Supplementary file 1 [file animals-15-02712-s001.zip › animals-3800986-supplementary.pdf]

PRISMA Flow Diagram

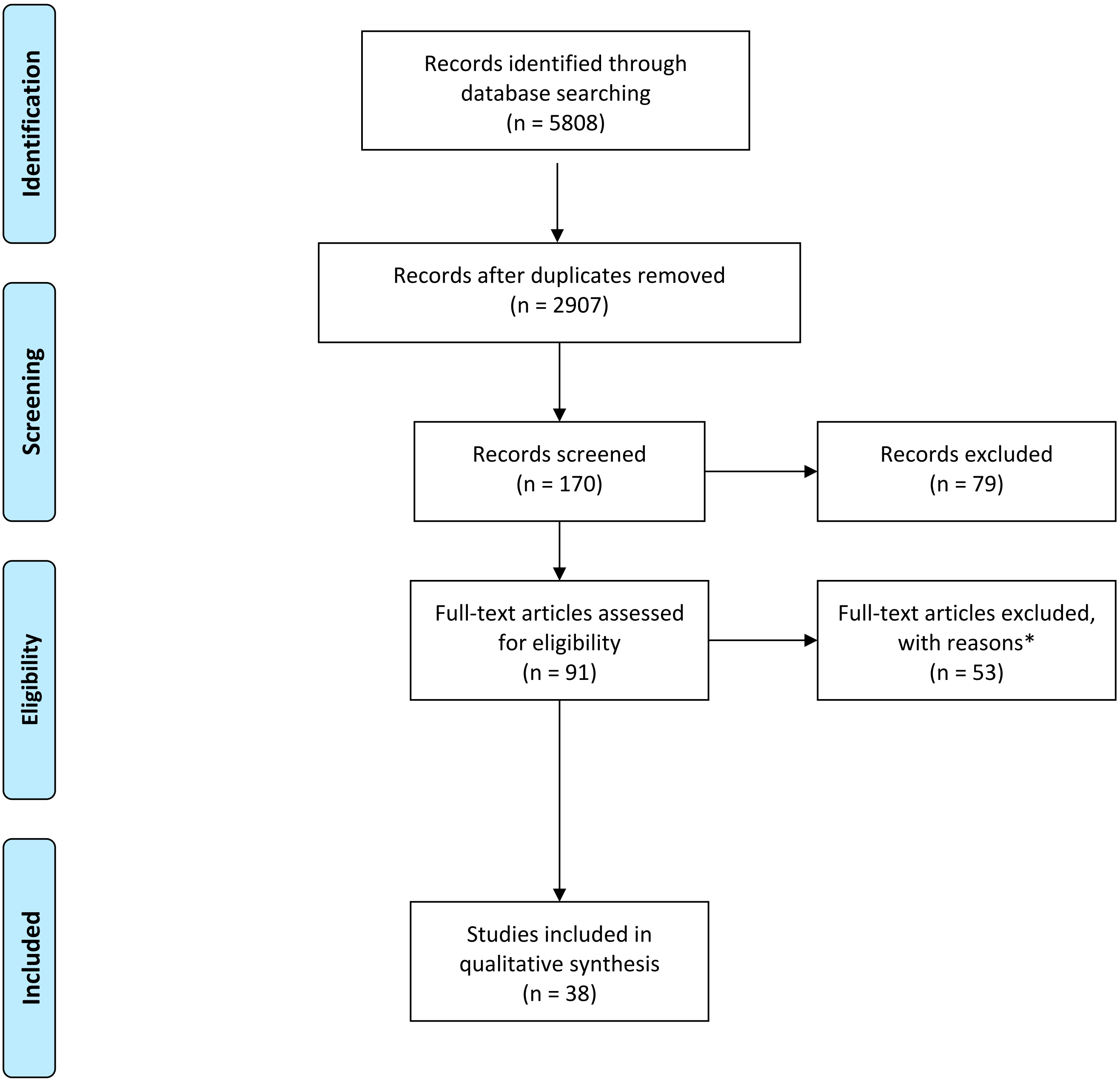

Slightly Modified From: Moher, D.; Liberati, A.; Tetzlaff, J.; Altman, D.G.; The PRISMA Group. Preferred Reporting Items for Systematic Reviews and Meta-Analyses: The PRISMA Statement. *PLoS Med.* **2009**, *6*, e1000097. https://doi.org/10.1371/journal.pmed.1000097.

**Reasons: Irrelevant topic, incorrect intervention, wrong animal model or setting, only abstract available*

For more information, visit www.prisma-statement.org.
